# Determining the functional significance of mismatch repair gene missense variants using biochemical and cellular assays

**DOI:** 10.1186/1897-4287-10-9

**Published:** 2012-07-23

**Authors:** Christopher D Heinen, Lene Juel Rasmussen

**Affiliations:** 1 Neag Comprehensive Cancer Center and Center for Molecular Medicine, University of Connecticut Health Center, 233 Farmington Avenue, ML3101 Farmington, CT, USA; 2 Center for Healthy Aging, University of Copenhagen, Blegdamsvej 3B, 2200 Copenhagen, Denmark

**Keywords:** Lynch syndrome, Hereditary nonpolyposis colorectal cancer, Mismatch repair, Variants of uncertain significance, MSH2, MSH6, MLH1

## Abstract

With the discovery that the hereditary cancer susceptibility disease Lynch syndrome (LS) is caused by deleterious germline mutations in the DNA mismatch repair (MMR) genes nearly 20 years ago, genetic testing can now be used to diagnose this disorder in patients. A definitive diagnosis of LS can direct how clinicians manage the disease as well as prevent future cancers for the patient and their families. A challenge emerges, however, when a germline missense variant is identified in a MMR gene in a suspected LS patient. The significance of a single amino acid change in these large repair proteins is not immediately obvious resulting in them being designated variants of uncertain significance (VUS). One important strategy for resolving this uncertainty is to determine whether the variant results in a non-functional protein. The ability to reconstitute the MMR reaction *in vitro* has provided an important experimental tool for studying the functional consequences of VUS. However, beyond this repair assay, a number of other experimental methods have been developed that allow us to test the effect of a VUS on discrete biochemical steps or other aspects of MMR function. Here, we describe some of these assays along with the challenges of using such assays to determine the functional consequences of MMR VUS which, in turn, can provide valuable insight into their clinical significance. With increased gene sequencing in patients, the number of identified VUS has expanded dramatically exacerbating this problem for clinicians. However, basic science research laboratories around the world continue to expand our knowledge of the overall MMR molecular mechanism providing new opportunities to understand the functional significance, and therefore pathogenic significance, of VUS.

## Introduction

Lynch syndrome (LS; also called hereditary nonpolyposis colorectal cancer, HNPCC) is a hereditary cancer susceptibility/predisposition disease caused by a heterozygous germ line mutation in the DNA mismatch repair (MMR) gene *MSH2MSH6MLH1* or *PMS2*[1,2]*.* Somatic loss or hypermethylation of the wild-type allele results in a cell with defective MMR [1-3]. Loss of MMR function likely leads to tumorigenesis through the establishment of a mutator phenotype that increases the likelihood of developing mutations in other oncogenes and tumor suppressors. The majority of MMR gene mutations currently detected in LS patients are assumed to be pathogenic as they result in deletion of the protein product. A significant problem, however, is the identification of an increasing number of germline missense variants in the MMR genes. Missense variants may account for 20-30% of mutations in LS patients [4,5], many of which are now catalogued in MMR gene mutation databases (e.g. http://www.insight-group.orghttp://www.mmruv.info). A causal role for the majority of these missense variants in disease pathogenesis is not immediately obvious, thus they are termed variants of uncertain significance (VUS). The identification of a deleterious germline MMR gene mutation provides a definitive diagnosis of LS, thus, the uncertainty of a VUS poses a major problem for clinicians and genetic counselors who must manage the patient and their family members.

Obtaining extensive clinical information about different VUS is important for determining those most likely to be pathogenic. Key information includes determining whether the variant segregates with the affected members in a suspected LS family, that the variant does not occur in >1% of the general population and that it associates with a tumor that displays hallmarks of defective MMR such as microsatellite instability (MSI)or loss of protein expression as determined by tissue immunohistochemistry (IHC). As the majority of LS-causing MMR gene mutations result in loss of protein expression, tumor IHC is a widely used first screen for diagnosing LS [6-8]. Certain VUS may affect protein stability, resulting in a negative IHC test, however, a VUS may affect MMR function without disrupting protein levels. Thus, it is also important to test whether the VUS affects MMR function through a variety of *in vitro* and cellular assays.

## A proposed decision tree for the analysis of MMR gene VUS

The use of functional assays makes up a significant portion of our previously proposed three-step diagnostic tree for assessing the pathogenicity of VUS in MMR genes [9]. Step 1 of this decision tree involves the current diagnostic procedure for suspected LS patients; analysis of the tumor phenotype by IHC and/or MSI testing, followed by testing for a mutation in the MMR genes. If a VUS is found, the diagnostic procedure continues to Step 2 which comprises *in silico* alignment- and splice site-based predictive analysis of the VUS. This includes the bioinformatic tool referred to as Multivariate Analysis of Protein Polymorphisms-Mismatch Repair (MAPP-MMR) which aides in the prediction of pathogenicity of *MSH2* and *MLH1* variants [10]. MAPP-MMR combines an analysis of the conservation of the altered amino acid with the change in physiochemical properties of the amino acid. The score derived from this algorithm allows for the classification of a given variant as neutral, deleterious or borderline.

Step 2 also includes widely used assays for measuring repair of mismatches *in vitro*. Due to work in basic science research laboratories over the past two decades our knowledge of MMR function has improved tremendously. The best characterized function of the MMR proteins is the ability to repair single base pair mismatches and small insertion/deletion loops (IDLs) [11-13]. The MMR pathway is initiated by the recognition of DNA lesions by a heterodimer of the MSH2 and MSH6 proteins which recognizes single base pair mismatches and small IDLs, or MSH2 and MSH3 which recognizes larger IDLs [14]. DNA mismatch recognition by MSH2-MSH6 stimulates an ATP for ADP exchange at adenosine nucleotide binding sites in both proteins resulting in the formation of ATP-bound MSH2-MSH6 sliding clamps on the DNA [15]. The sliding clamps recruit a second MMR heterodimer consisting of MLH1 and PMS2. MLH1-PMS2 binds several MMR proteins and modulates their activity in a mismatch-dependent manner. PMS2 harbors a latent endonuclease activity that when activated in a mismatch-dependent manner introduces a nick in the daughter strand, 5’ of the mismatch [16]. The exonuclease EXO1 loads at this nick in a MMR-dependent fashion and excises the misincorporation-containing DNA strand. Once excision extends past the site of the mismatch, the excised strand is resynthesized. Loss of MMR function leads to increased genomic instability which has been proposed to accelerate the accumulation of mutations in important oncogenes and tumor suppressors that drive tumorigenesis [17]. Evidence for a mutator phenotype in Lynch syndrome cancers is readily apparent, most noticeably through the increase of MSI which includes frameshift mutations at small repeat sequences in known cancer-associated genes from MMR-defective cancers [18].

The repair of mismatches has been reconstituted *in vitro* providing valuable information about the proteins necessary for MMR and their roles during the process [19-21]. Cellular extracts or recombinant MMR proteins are tested for their ability to repair a DNA plasmid that contains a single mismatch within an endonuclease restriction site. This repair assay also has been utilized to study the repair capabilities of VUS-containing MMR proteins as reviewed previously [22,23]. Assessing the repair of mismatches is likely the most biologically relevant assay for assessing the function of a variant. However, the preparation of reagents is tedious and the assay itself requires certain technical specialization. Recently, a cell-free assay for testing the MMR function of *MLH1MSH2* and *MSH6* variants was described [24,25]. This system utilizes PCR mutagenesis and *in vitro* transcription and translation to generate VUS-containing MMR proteins for use in the *in vitro* repair assay. This approach eliminates the time consuming and technically difficult cloning and protein purification steps which begins to make these tests more amenable for clinical diagnostic labs. However, before this assay can be used in clinical diagnostic laboratories it needs to be validated.

## Step 3 assays for assessing VUS function

**Figure 1:**
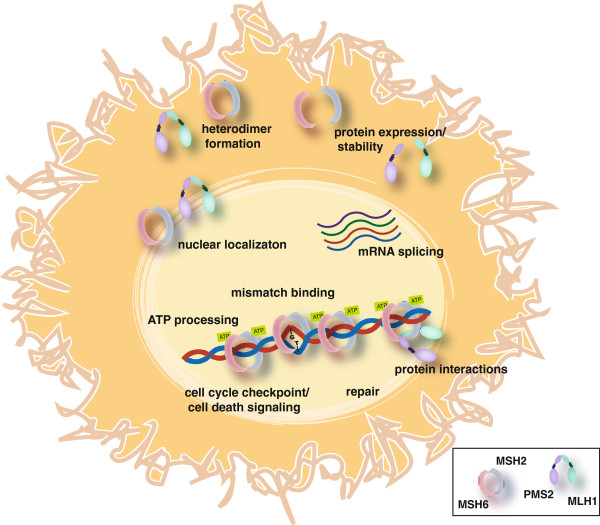
**Mismatch repair functions tested in Step 3 assays.** Step 3 assays have been designed to test the effects of cancer-associated VUS in various aspects of mismatch repair function. These assays test VUS effects on mRNA splicing, protein expression levels, ability to interact with other mismatch repair proteins including known heterodimer partners, cellular localization, DNA mismatch binding, ATP binding and hydrolysis, repair and cell cycle checkpoint and apoptosis activation in response to certain mutagenic reagents.

If the results after Step 2 are inconclusive, the VUS remains unclassified. An additional layer of diagnostic assays can include an examination of other cell-biological, biochemical and biophysical analyses of the MMR proteins (Step 3). These assays often address more specific aspects of the protein function beyond measuring repair of mismatches (Figure 1).

### *In vitro* biochemical assays

The formation of heterodimers between the MMR proteins is key to their function. *In vitro* approaches have been utilized to examine heterodimer formation between MSH2 variants and a GST-tagged MSH3 or MSH6 revealing that none of the variants tested affected heterodimer formation [26]. Similarly, the same group examined the ability of MLH1 variant proteins to bind to its partner PMS2 [27]. In this study, 10 of the 11 variants analyzed had a detrimental effect on heterodimer formation.

In addition to heterodimer formation, *in vitro* assays have been used to test the effects of VUS on discrete biochemical steps in the MMR molecular mechanism. Our group has studied recombinant MSH2-MSH6 carrying single amino acid alterations in MSH2 or MSH6 for their ability to perform several biochemical functions including mismatch binding and ATP hydrolysis [28-30]. As MSH2-MSH6 must bind and hydrolyze ATP to execute MMR, a variant that is deficient in these activities is very likely pathogenic. Similar studies have examined the ability of the VUS containing heterodimer to bind to mismatched DNA and release mismatches in an ATP-dependent manner; both functions critical to proper MMR [31-35]. However, the assays performed in these studies are not readily transferrable to a clinical diagnostic lab as they require extensive specialization. The value of these studies mainly lies in the basic science research lab where the variants may be used as tools to improve our overall understanding of MMR.

### Cell-based assays

The main limitation of the *in vitro* biochemical assays described, including the repair assays, is that they do not exactly recapitulate the environment in which the MMR proteins function and, therefore, may not fully reflect how the variant will act inside the human cell. For example, the MMR proteins need to identify and repair mismatches in the context of chromatin, most likely in cells undergoing active DNA replication. The manner in which the MMR proteins interact with chromatin is only beginning to be understood [36-38] and, therefore is difficult to model *in vitro*. Studies of the MMR variants in the context of the human cell will be essential to fully understand their effect on MMR function. The earliest attempts to examine VUS function in human cell culture models utilized transfection to re-express the VUS in a MMR null background [39]. Expression of *MLH1* VUS in 293T human embryonic kidney fibroblasts revealed that some of the variant containing proteins were expressed at lower levels than a wild-type control which may suggest an effect of the VUS on transcript or protein stability. A more recent study demonstrated a reduced protein-half life for two *MLH1* VUS transfected into HCT-116 colorectal cancer cells indicating that the reduced expression observed was due to decreased protein stability [40]. However, a large-scale study of *MLH1* VUS transfected into HCT-116 cells concluded that protein expression levels do not always correlate with repair capability [41]. Extracts from the transfected cells were examined for protein expression levels and then used in *in vitro* MMR assays. Some VUS were expressed at low levels, yet the extracts displayed restored repair function. Transient transfections were also used to determine that an L749Q variant in MLH1 disrupted interactions with its heterodimer partner PMS2 [40]. Disrupted interaction with PMS2 is assayed by measuring the level of PMS2 protein in the cell. The PMS2 protein is unstable if it is not in complex with MLH1 and, therefore, reintroduction of functional MLH1 will lead to restored PMS2 protein levels as well [42-44]. A similar study of *MSH2* VUS transfected into MSH2-null LoVo colorectal cancer cells was performed to detect effects of VUS on MSH2-MSH6 interactions [45]. Like PMS2, MSH6 protein is unstable when not in complex with MSH2 [46]. All 15 VUS studied resulted in near wild-type levels of protein expression and normal interaction with MSH6.

There are several challenges when attempting to complement human cell lines in culture. The first challenge is selecting a cell line to use. To isolate the contribution of the variant-containing MMR protein, cell lines that lack expression of the endogenous MMR protein are most useful. While it is possible to use small inhibitory RNAs (siRNA) to specifically knock-down the levels of the endogenous protein prior to introducing an siRNA-resistant variant transgene, these studies are tedious and may lead to misinterpretations as complete elimination of the endogenous wild-type protein is unlikely. Thus, the cell lines most commonly used are human cancer cell lines that have suffered mutational inactivation of the endogenous MMR genes. Previous studies have identified cancer cell lines that lack MSH2, MLH1 or MSH6 expression where MMR functions can be restored by re-introduction of the wild-type gene [47-50]. Cancer cell lines generally grow well in culture and are immortal which makes it easy to generate large numbers of cells for performing biochemical assays. However, the genetic background of these lines is uncertain and likely unstable possibly masking the function of some VUS. Some studies have avoided the need to use MMR-null cancer cell lines by adding a protein tag to the variant transgene to distinguish it from endogenous wild-type protein [32,51,52]. Through use of fluorescent protein fusions, we previously were able to track expression and cellular localization of the variant proteins in NIH-3T3 primary mouse embryonic fibroblasts (MEFs) (discussed further below) [32]. This approach is limited to those assays in which the tagged protein can be isolated from the endogenous wild-type protein. In addition, the presence of a large protein tag may influence the function of the MMR protein, which needs to be examined in carefully controlled experiments [53].

Another challenge to introducing MMR VUS into human cells is the method of delivery. Transient transfection, while the easiest approach, presents complications. Certain cell lines do not transfect very efficiently, and the nature of the method often results in heterogeneity with regards to the levels of exogenous protein expressed. Obtaining accurate levels of MMR protein expression is likely very important for assessing function. Overexpression of MSH2 or MLH1 appears to be toxic to some cells [54], while underexpression may result in limited function [50,55]. One approach to address the delivery problem is to directly target the MMR gene locus in cells to “knock-in” the variant allele. A recent study took advantage of the extensive knowledge of gene targeting in mouse embryonic stem cells to generate “knock-in” alleles for four *MSH2* VUS [56]. The authors generated homozygous mutant cells that were tested for protein expression and MMR function. The advantage of this approach is that the VUS are expressed from the endogenous promoter allowing for normal regulation of gene expression and mRNA processing. Additionally, the mouse embryonic stem cells can be used to generate mice to directly assess the effect of the VUS on tumorigenesis. The major disadvantages are the technical difficulty of the approach and the fact that these studies are performed in mouse cells using the murine MMR genes. Though gene targeting has generally been very difficult in human cells in culture, the use of recombinant adeno-associated viruses [57], zinc finger nucleases [58] and transcription activator like effector nucleases (TALEN) [59] for targeting has made these studies more feasible in recent years.

### Nuclear localization

One aspect of VUS function that can only be studied in cellular assays is whether it affects localization of the protein. Nuclear import of the MMR proteins is obviously a prerequisite for proper DNA repair. For that reason, nuclear translocation presents an additional regulatory mechanism to both protein expression and protein-protein interactions that could be disrupted by a VUS. Moreover, the stoichiometry of repair complexes is essential for efficient DNA repair indicating that both the expression and nuclear translocation of DNA repair proteins must be tightly regulated in order to maintain the genomic integrity [60]. We have previously examined the consequences of seven *MSH2* VUS found in LS families by expressing the variant cDNAs fused to a fluorescent tag as described above. We show that two variant proteins, MSH2-P622L and MSH2-C697F affect nuclear localization in the cell while also conferring *in vitro* biochemical defects, namely in mismatch binding, and *in vivo* interaction defects with MSH6 and EXO1 as measured by yeast two-hybrid assays [32].

### The MMR-dependent DNA damage response

The other major advantage to testing MMR VUS in the context of the cell is the ability to examine other MMR functions in addition to repair of single basepair mismatches. The MMR proteins are also involved in the activation of cell cycle checkpoints and apoptosis in response to certain DNA damaging agents. MMR-deficient tumor cell lines and MEFs from *Msh2Mlh1* and *Msh6* knockout mice are more resistant to treatment with certain DNA damaging agents such as cisplatin and N-methyl-N’-nitro-N-nitrosoguanidine (MNNG) [54,61-68]. We have previously proposed that the loss of this damage response in MMR defective cells may provide cells a temporary selective advantage in an environment conducive to increased DNA damage [69,70]. Thus, loss of the MMR-dependent damage response may play a role, along with the mutator phenotype, in tumorigenesis. Consistent with this theory, mice carrying a missense mutation in *Msh2* that appeared to disrupt DNA repair while maintaining damage response functions, displayed tumor onset that was significantly delayed compared to *Msh2*-null animals [71]. In a second study of mice with a dominant missense mutation in *Msh6*, a similar result was observed [72]. These results suggest that both MMR-mediated checkpoint/apoptosis response and DNA repair affect tumorigenesis. Thus, understanding how VUS affect the MMR-dependent damage response may be important for determining their contribution to tumorigenesis. In addition, this information may be important for predicting how VUS carriers may respond to certain therapy. Multiple studies have suggested that patients with MMR-deficient tumors do not benefit from some commonly used chemotherapies such as 5-fluoruracil, a common component of colorectal cancer treatment regimens [73-76]. We recently examined four *MSH2* VUS for their ability to restore repair and damage response functions to the MSH2-null Hec59 endometrial cancer cell line [77]. The VUS were stably introduced into cells through use of a lentiviral expression vector which allowed us to more carefully control for expression levels as well as examine the function of the variant protein over multiple cell generations. We observed that two of the four VUS restored both repair, through use of an *in vivo* MMR assay [78], and response to MNNG including cell survival and cell cycle checkpoint activation. Similarly, Wielders et al., examined response to MNNG in their mouse embryonic stem cells carrying *MHS2* VUS and determined that cells carrying the P622L variant caused increased MSI and failed to respond to the drug [56]. Transient transfection of four *MLH1* VUS into the MLH1-defective A2780 ovarian carcinoma cells failed to restore *N*-methyl-*N*-nitrosourea sensitivity, whereas transfection with two known *MLH1* polymorphisms reversed the methylation tolerant phenotype [79].

### Studying VUS in yeast

The effects of variants on MMR function have also been examined *in vivo* in yeast [33,35,80-86]. The relative ease of gene targeting in yeast makes the creation of strains carrying different MMR gene variants feasible. These variant strains can be tested for repair of marker genes carrying homopolymeric repeats. *msh2Δ* yeast display a 290-fold increase in repeat tract instability compared to wild-type yeast. Re-expression of *MSH2* restores repair levels, however an examination of 7 missense variants of *MSH2* showed that all 7 failed to restore normal repair activity [34]. Similarly, another study identified 33 of 54 *MSH2* VUS that fail to restore repair in an *msh2Δ* strain compared to wild-type control [79]. A study of *MLH1* variants in yeast demonstrated 15 out of 28 that were defective for repair activity [82]. However, in a similar assay in a different strain of yeast, the same variants displayed different repair capabilities suggesting that genetic background may affect the function of some VUS. As genetic background effects will certainly not be limited to yeast, these results show the importance of *in vivo* approaches to studying variant function. The relative ease of yeast genetics allows one to readily test multiple variants and the repair assays utilized are straight-forward and quantitative. The assays can be standardized between labs, though likely those labs will already need to be highly trained yeast laboratories. One major disadvantage of this approach is that only those variants which are conserved across species can be tested. To address this disadvantage, researchers have made attempts to “humanize” the yeast genome by creating hybrid human-yeast MMR genes [86,87] or cloning in an entire human MMR gene into the yeast locus [88]. These approaches were then used to examine the functionality of VUS in *in vivo* MMR assays. Another inescapable disadvantage is that despite the similarities in MMR mechanism between yeast and humans, there may still be important differences that affect interpretation of yeast-based assays. Post-translational modifications, protein-protein interactions and other aspects of MMR regulation may differ in yeast and could affect VUS-containing protein function. Clinicians often are hesitant to rely on VUS functional data from non-mammalian systems for diagnostic purposes without corroboration from studies with human cells and proteins.

### RNA splicing

In addition to affecting protein function, VUS have also been shown to have effects on mRNA splicing by altering exonic splicing regulatory sequences. Analyses of VUS-containing *MSH2* or *MLH1* mRNA from patients revealed splicing defects that included exon skipping and intron inclusion [89,90]. Step 2 of our proposed decision tree involves the use of *in silico* predictions to identify VUS that may affect splicing. These identified variants can be further tested in cell culture experiments that involve transfecting the relevant portion of the patient genomic DNA cloned into a splicing reporter minigene [91,92]. It is important to note that silent mutations as well as nucleotide changes in intronic sequences can also affect splicing, widening the scope of VUS that need to be considered.

## How much function is not enough and other challenges

Though determining a genotype/phenotype relationship for VUS should provide clear evidence for contribution to disease, there are several challenges that keep it from being so straight forward. VUS that result in loss of MMR activity similar to that observed when the protein is absent can most likely be considered pathogenic. However, many VUS examined show intermediate activity that, while significantly increased over null controls, does not match the level of the wild-type protein. In our ATP hydrolysis studies, we identified some variants that failed to function much above background, however, the majority of VUS demonstrated intermediate activity [28-30]. This is not limited to Step 3 assays, as VUS displaying intermediate activity have been observed in many of the studies examining repair function as well [24,39,41,45,93]. The challenge is determining the significance of these intermediate functional effects on disease phenotype. Such subtle defects in protein function may arise from weak disease alleles that, through a modest reduction in repair efficiency, can increase genomic instability and contribute to tumorigenesis. However, the expected mutator phenotype in these cells would be milder slowing the accumulation of tumor-causing mutations and resulting in reduced penetrance compared to stronger LS-causing alleles. Presentation of disease in carriers of weak alleles may depend on the genetic background in the patient. The functional defect of some VUS may not be sufficient to efficiently drive cancer formation on its own, but combined with other alleles that affect DNA replication fidelity would generate the levels of genomic instability necessary to drive tumorigenesis. Studies of different MMR VUS modeled in yeast suggest that polygenic interactions are possible between two weak alleles that combine to produce a stronger MMR defect [94]. These authors conclude that the low penetrance of certain MMR VUS may be due to the fact that additional mutational “hits” in other MMR genes are required in the tumor to generate the strong MMR defects necessary to drive disease. Consistent with this model, a recent study described an association between germline variants of *MSH3* and an *MSH2* VUS in an LS family [95]. Members of the family who carried both the *MSH3* and *MSH2* variants developed early-onset colon tumors marked by MSI. However, those family members that carried only the *MSH3* variant or the *MSH2* VUS alone did not develop LS tumors.

The interpretation of these intermediate results will improve as more studies are performed. Future studies will need to include more established strong disease alleles or clear polymorphisms (as determined by clinical and genetic information) as positive and negative controls in order to define the range of functional activity associated with disease. In addition, as more studies are performed, the likelihood that certain VUS will be tested in multiple assays increases. We already can begin to compare results for some VUS analyzed by different approaches [96]. For example, we previously determined that the *MSH2* D167H and K393M variants had intermediate effects on MSH2-MSH6 *in vitro* adenosine nucleotide processing [29]. More recently, we expressed these same two variants into an MSH2-deficient cancer cell line and observed that they restored cellular MMR functions, including repair of a G/T mismatch and response to alkylation damage, to near wild-type levels [77]. These results suggest that the biochemical defects associated with D167H and K393M are not sufficient to contribute to disease. However, an *MSH2* P622L variant, which has been shown to more dramatically disrupt MSH2-MSH6 *in vitro* biochemical function [29,32,35], displays clear cellular MMR defects when expressed in yeast [35,79,81] or mammalian cell culture systems [32,77]. By comparing *in vitro* and *in vivo* approaches, we can begin to determine what level of biochemical activity is associated with an inefficient cellular damage response. Multiple studies of the same VUS do not always result in clear interpretations, however. Reintroduction of the *MLH1* R265C variant into MLH1 null 293T cells resulted in normal expression levels and restoration of repair function [39]. However, studies expressing the variant in HCT116 cells demonstrated reduced protein stability [40] and only intermediate repair (55% compared to 79.7% for wild-type) [41]. A fourth study reported that the R265C UV failed to restore MMR activity at all in MLH1 deficient cell extracts [97]. Finally, introduction of the equivalent variant into yeast resulted in a strain that demonstrated an intermediate mutator phenotype compared to the wild-type strain indicating a defect in repair [83]. The conflicting results suggest that VUS may have different activities depending on the assay system, the genetic background of the cell line utilized or other variables between laboratories. Thus, caution must be used when relying on a single functional study to interpret disease significance.

## Conclusions

Despite the challenges described above, the recent development of second-generation assays holds promise for the development of a widely applicable diagnostic procedure. Increased functional testing from multiple laboratories will allow for improved standardization of techniques as well as strengthen our ability to interpret the results, particularly for those VUS with only intermediate effects. However, functional studies of the variant-containing protein should be only one component of a multiple-step analysis of a VUS when determining pathogenicity. In addition to examining VUS in multiple functional assays, these results should be combined with available clinical data such as segregation with disease in families and absence of the variant in control populations. Other tumor features may be informative as well, such as somatic loss of the remaining wild-type allele in the tumor and certain morphological or histopathological features characteristic of LS cancers [98]. With improving technologies and falling prices, sequencing of the MMR genes may become more common in patients. Initially, this may occur as part of a universal screening of all CRCs and eventually in the context of routine genome sequencing that may become a greater part of personalized medicine. Likely, this means that the MMR VUS problem will continue to grow. However, the increased attention being paid to this problem by clinicians and scientists increases the likelihood that we will be able to more accurately classify these variants in the near future. A validated functional test could be used to help diagnose LS in those patients carrying a MMR gene VUS even in the absence of strong family historyinformation.

## Abbreviations

LS, Lynch syndrome; VUS, Variants of uncertain significance; MMR, Mismatch repair; MSI, Microsatellite instability; IHC, Immunohistochemistry; MAPP-MMR, Multivariate Analysis of Protein Polymorphisms-Mismatch Repair; IDLs, Insertion/deletion loops; TALEN, Transcription activator like effector nucleases; MEF, Mouse embryonic fibroblasts; MNNG, Methyl-N’-nitro-N-nitrosoguanidine.

## Competing interests

The authors declare that they have no competing interests.

## Authors’ contributions

Both authors contributed to the conception, design, data analyses and manuscript preparation. Both authors read and approved the final manuscript.
